# What is the best algorithm for investigation of acute lower gastrointestinal haemorrhage? 

**Published:** 2017

**Authors:** Ainkaran Santhirasekaram, Sherif Latif, Easha Arooj, Kamran Rostami, Sauid Ishaq

**Affiliations:** 1 *Department of Radiology, Russell Hall Hospital, Dudley, DY1 2HQ, UK*; 2 *Department of Gastroenterology, Russell Hall Hospital, Dudley, DY1 2HQ, UK*; 3 *Gastroenterology unit Milton Keynes University Hospital, UK *; 4 *Birmingham City University, Birmingham, UK *; 5 *SGU, Grenada West Indies, UK *

**Keywords:** Lower gastrointestinal (GI) bleed, Colonoscopy, CT angiogram, Catheter angiography.

## Abstract

An 81-year-old male presented with multiple episode of severe PR bleeding over 2 days. CTA done prior to catheter angiography that enabled successful intervention. This case emphasises the importance of CTA prior to catheter angiography in acute LGIB

## Introduction

Because of therapeutic benefit, traditionally it is recommended to have a colonoscopy in patients with a lower GI bleed who are haemodynamically stable. In a patient who is not haemodynamically stable one would consider emergency surgery or catheter angiography. However, it is not clear in guidelines whether it would be essential to organise a CT angiogram (CTA) prior to these procedures in patients with an acute lower GI bleed. While there are studies which recommend the use of a CTA, there are other studies against using CTA in these circumstances. Here we present a case with an acute lower GI bleed who became haemodynamically unstable. In this case study, we show that the catheter angiogram is not able to identify the site of bleeding without the aid of the CTA prior.

## Case Report

An 81-year-old male presented with multiple episode of severe PR bleeding over 2 days. He denied any nausea or vomiting and any change to his bowel habit. He had no history of any PR bleeding and has no significant past medical history. He was not currently on any medication. On examination, his abdomen was distended and non-tender. DRE showed bright red blood but no palpable masses. He was tachypnoeic with a respiratory rate of 31 breaths per minute and his BP was dropping from 151/90 to 130/88 and his heart rate increased from 88 bpm to 98bpm 2 hours into his admission. His Hb was 69 gm/L (12.0-15.5 g/L), lactate; 2.00 mmol/L (normal 0.5-1 mmol/L), urea; 30.5 mmol/L (normal 3.5-6.5 mmol/L), creatinine; 444 mmol/L (normal 60-120 micromole/L) and INR was 1.0 (normal<1.2) on admission. All other bloods were normal and his ABG showed compensated metabolic acidosis. He was initially treated with IV fluids and transfused 4 units of red blood cells. He was started on IV pantoprazole and IV tranexamic acid.

**Investigations and management**

Initially an OGD was performed which showed a hiatus hernia, gastritis and duodenitis but no acute upper GI bleed was found. At this point the patient was becoming more haemodynamically unstable with the blood pressure dropping to 109/79 from 130/88. A colonoscopy was not advised due to the patient acutely unwell and bowel not being prepared that would make it unsafe to carry out the procedure and technically difficult to locate the bleeding point. With the high rate of bleeding patient became unstable, hence colonoscopy was not considered and the patient sent immediately to have a CT angiogram. 

**Figure 1:**
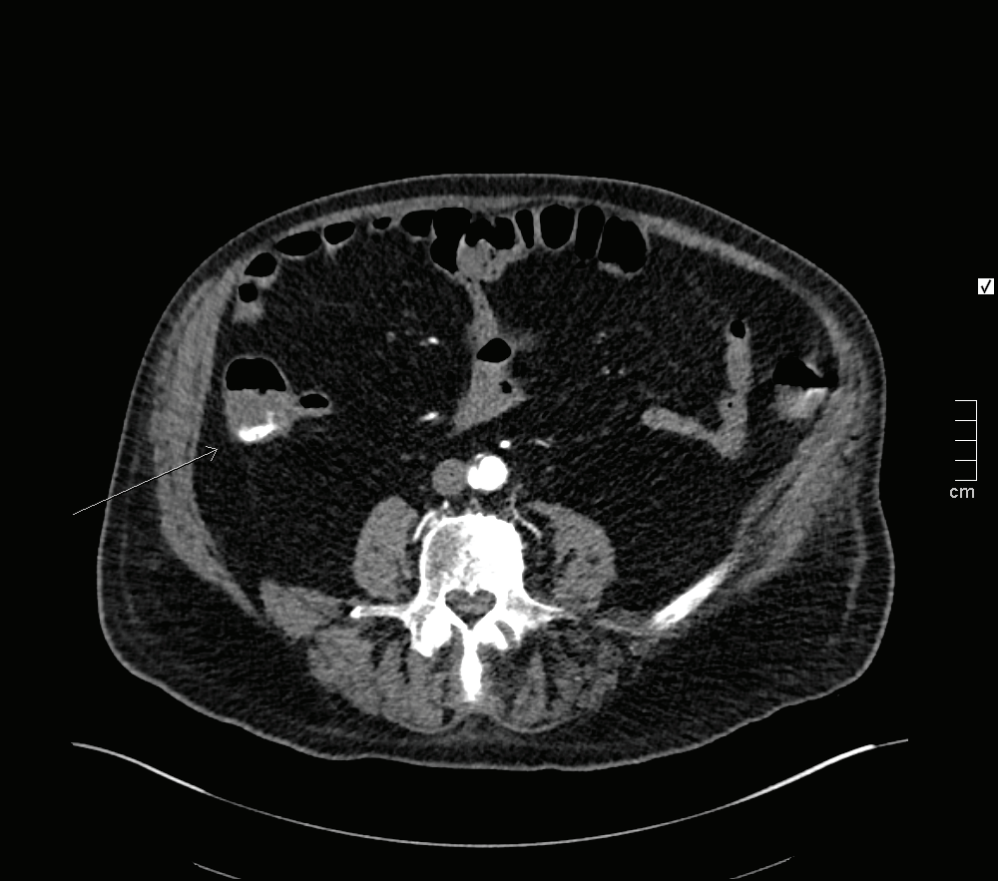
CT angiogram demonstrating active contrast extravasation in the caecum (arrow

According to figure 1, active extravasation in caecum appears to be supplied by the ileo-colic artery, and no mass lesion was present, which could be due to angiodysplasia.

**Figure 2:**
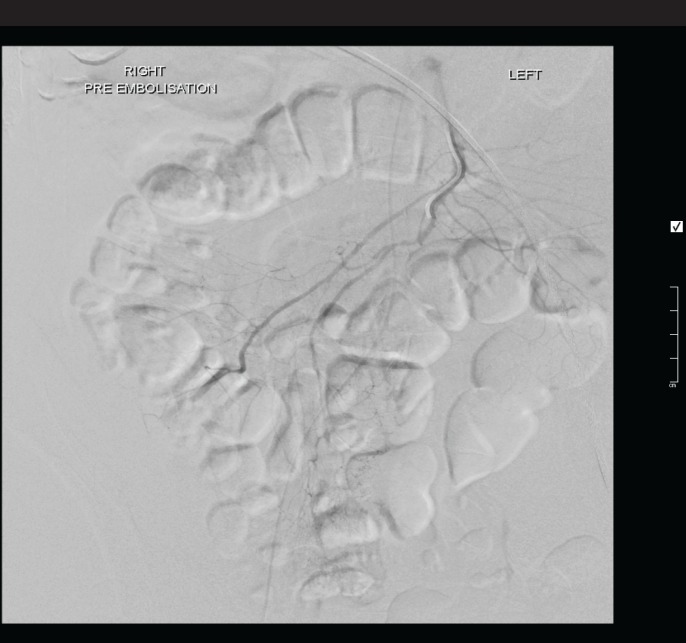
SMA catheter angiogram did not demonstrate any active bleeding

**Figure 3:**
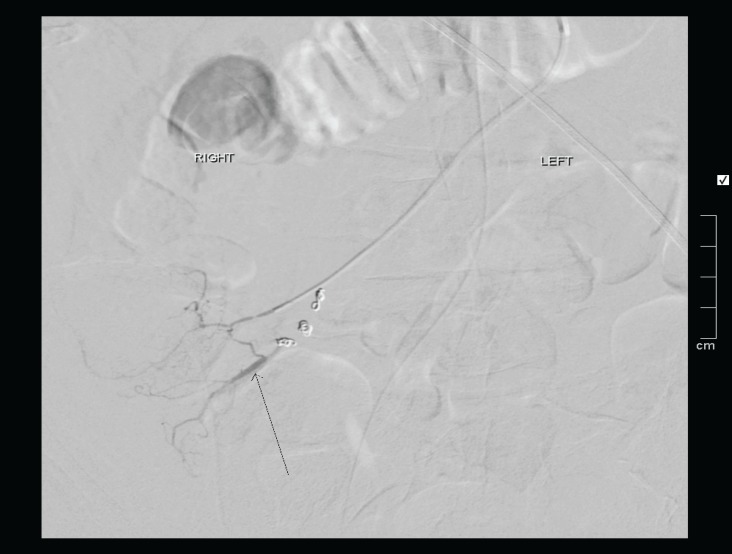
As the bleeding point was known to be from the caecum on CTA, a microcatheter was advanced into distal branches of the ileocolic artery and microcoils placed. After some coils were placed the branch demonstrating the bleeding point was identified (arrow

**Figure 4:**
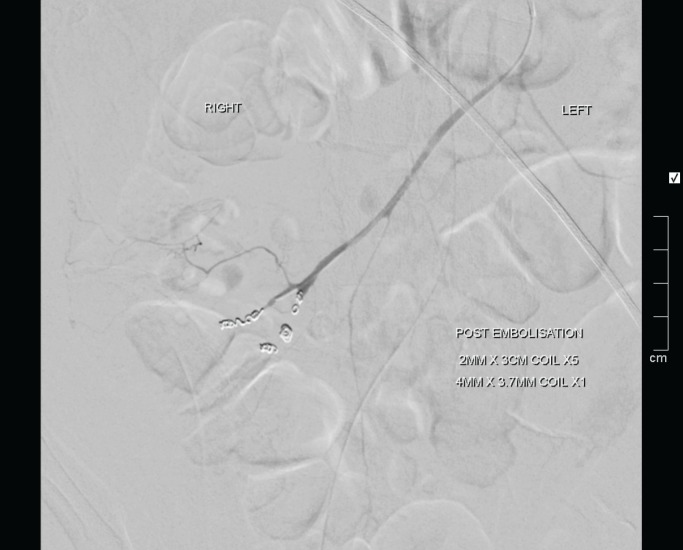
final angiogram after a successful embolization of the bleeding point which emanated from distal branches of the ileocolic artery

The patient was immediately taken to the angiography theatre for catheter angiography. The coeliac angiogram was firstly negative and the bleeding point was not visible on the superior mesenteric artery angiography possibly due to spasm, as noted on the below catheter angiography (fig.2). A microcatheter was therefore placed into the distal ileocolic artery where the bleed was noted on branches of the ileocolic artery as shown below (fig.3). The bleed was successful coiled with microcoils (fig.4**)**.

The patient had no further episodes of PR bleeding and was discharged with a stable Hb of 106. He was commenced on a 5-day supply of tranexamic acid,a 1ferrous sulphate and folic acid on discharge.

## Discussion

A lower GI bleed (LGIB) is defined as blood loss originating distal to the ligament of Treitz and accounts for 20-30% of all major GI bleeds [1]. LGIB typically presents as haematochezia. In practice 80-85% of LGIB originates distal to ileocaecal valve, with only 0.7-9% from the small intestine. The remaining 10-15% of cases from the upper gastrointestinal tract present as brisk bleeding rather than melaena. The severity of the bleeding varies greatly from a small amount to a life threatening haemorrhage. The definition of an acute GI bleed is a GI bleed which lead to haemodynamic instability or needs a blood transfusion [1]. Lower GI bleeds most often arises from the colon or anorectum and very rarely from the small bowel [2]. Ninety percent of lower GI bleeds resolve spontaneously and has a mortality rate of only 4% [2]. The commonest cause of a lower GI bleed is colonic diverticulosis which accounts for 30-40% of cases [3]. Vascular ectasia similar to this case is the second most common cause and accounts for around 10 % of the lower GI bleeds aetiology [3]. Other causes of lower GI bleed include iatrogenic causes, malignancy, IBD, ischemic colitis, anorectal abnormalities and other rarer causes [3]. 

The key differential diagnosis one needs to be aware of is an upper GI bleed; hence all patient with who present with bright red rectal bleeding and are in shock should have urgent gastroscopy even without hematemesis. Thirteen percent of GI bleeds occur just proximal to the ligament of Treitz and these patients are often haemodynamically unstable [4]. Therefore, it is essential for the patient to have an upper GI endoscopy as the consequence of overlooking an upper GI bleed in someone who is haemodynamically unstable can be very serious. This patient had an upper GI endoscopy to rule out a GI bleed.

The first step to assessing a GI bleeding is to establish a clear clinical history and examination to deduce whether this is an acute bleed and needs immediate intervention as well as assessing the cause of the bleed. It is then necessary to order the correct blood tests to determine whether this patient needs a blood transfusion and needs to be NBM. Afterwards one needs to order the correct imaging and scoping investigations from both a diagnostic and therapeutic point of view.

Traditionally, colonoscopy remains first line for lower GI bleeds in haemodynamically stable patients due to its ability to precisely locate the site of bleeding and apply therapeutic intervention [5]. It however has the disadvantage of being unable to see the small bowel and having poor visualisation in an unprepared bowel which would makes its use in an unstable patient who needs immediate intervention to be less useful [5]. In fact, the sensitivity to detect the source of the bleed drops to 21% in an unprepared bowel [5]. Rapid bowel preparation with 3-4 litres of polyethylene glycol and metoclopramide as a pro-kinetic agent can be effective [6]. Yet, there is a concern regarding the fluid overload in an acutely unwell patient. It is also considered unsafe to have sedation during colonoscopy in an acutely unstable patient [7]. Additionally, in cases of pain with PR bleeding, one needs to be concerned about ischemic colitis in which case a CT angiography could be a more appropriate investigation [6]. Some studies suggest that colonoscopy can be performed on 95% of patients with the lower GI bleed and a clear source of bleeding can be identified in 75-80% of cases while other studies reported lower rate of identification the bleeding source. [6]. In this patient where angiodysplasia is thought to be the most likely cause of bleeding, colonoscopy might have a sensitivity of 80% to detect angiodysplasia in comparison to CTA which has a sensitivity of 70% and specificity of 100% [8]. Additionally, studies are showing that emergency colonoscopies in an acute lower GI bleed leads to shorter hospital stays, however more prospective studies are needed to ascertain the usefulness of colonoscopy in an acute setting [9]. 

The CT angiogram can detect bleeding rates which are between greater than 0.5mls per minute and has a sensitivity and specificity rate of 90-99%, respectively in a massive lower GI bleed [10]. Traditionally, the CTA is indicated when one is actively bleeding but is haemodynamically stable [11]. It has the advantage of being non-invasive and able to detect the location of the GI bleed more accurately and target treatment such as embolization [5]. Also, CTA has the advantage of providing a 24-hour service unlike a colonoscopy [5]. Also, compared to angiography it can detect any soft tissue changes and perhaps even detect an upper GI bleed. However, the disadvantages of CTA include its unreliability to detect a bleed in an intermittent bleed and chronic bleeding [6]. A colonoscopy is therefore a better choice in intermittent bleeding or chronic bleed or acute bleed when the patient is stable. CTA is contraindicated in those with AKI or CKD [5]. 

Radionuclide imaging (RNI) uses technetium (99mTc) sulphur colloid which has a short half-life and 99mTc pertechnetate which has a longer half-life [12]. It has the advantage of being more sensitive to detect a GI bleed as low as 0.1ml per minute [5]. Its sensitivity is in fact around 94 percent in an acute lower GI bleed, but it is less specific (95%) than the CTA [12]. This makes its use more valuable in an intermittent GI bleed or when a bleed is not identified in a CTA, angiography and colonoscopy. Its main disadvantage is that it can only locate the GI bleed to an anatomical location and cannot identify the bleed to a specific part of the colon like the CTA [12]. This makes the use of RNI for planning emergency therapeutic intervention futile. In this case patient was unstable, hence radionuclide scan not considered as it would have delayed intervention. CTA provided useful information that the bleed most likely originate from the ileocolic artery which would not be provided by radionuclide imaging.

Catheter angiography (CA) is usually reserved for those patients with haemodynamic instability in whom colonoscopy is not appropriate, or those with persistent and recurrent bleeding [13].

CA may detect bleeding with rates above 1ml per minute compared to CTA [6].

Although most bleeds from diverticulae or angiodysplasia receive their blood supply from the superior mesenteric artery (SMA), when looking for a bleeding source many radiologists favour catheterising the inferior mesenteric artery (IMA) first as to avoid a bladder full of contrast, which often obscures the inferior mesenteric branches [14]. If the patient has already undergone CTA or has had the site marked with a clip at endoscopy, then localisation is made much easier as in our case.

The advantage of catheter angiography is its ability to accurately identify the bleeding source and apply therapeutic intervention like embolise with coils or gel. It has a sensitivity rate to locate a bleed of 46-82% which is much lower than a CTA and specificity of around 100% [5]. 

A CTA prior would have a higher sensitivity in detecting a lower GI bleed. Therefore, we recommend CTA as the first line in patient with LGIB who are haemodynamically unstable followed by CA if bleeding source detected.

**Learning Points**

This case emphasises the importance of CTA prior to catheter angiography in acute LGIB.

Colonoscopy is still recommended as a first line investigation in patients with LGIB who are haemodynamically stable.

## References

[1] Raju GS, Gerson L, Das A, Lewis B (2007). American Gastroenterological Association American Gastroenterological Association (AGA) Institute technical review on obscure gastrointestinal bleeding. Gastroenterology.

[2] Bounds BC, Friedman LS (2003). Lower gastrointestinal bleeding. Gastroenterol Clin North Am.

[3] Reinus JF, Brandt LJ (1994). Vascular ectasias and diverticulosis Common causes of lower intestinal bleeding. Gastroenterol Clin North Am.

[4] Strate LL (2005). Lower GI bleeding: Epidemiology and diagnosis. Gastroenterol Clin North Am.

[5] Kim BS, Li BT, Engel A, Samra JS, Clarke S, Norton ID, Li AE (2014). Diagnosis of gastrointestinal bleeding: A practical guide for clinicians. World J Gastrointest Pathophysiol.

[6] Jensen DM, Machicado GA (1988). Diagnosis and treatment of severe hematochezia The role of urgent colonoscopy after purge. Gastroenterology.

[7] McArdle K, Leung E, Latif S, Bohra A, Ishaq S (2010). Management of lower gastrointestinal bleeding: Endoscopist or radiologist?. Gut.

[8] Höchter W, Weingart J, Kühner W, Frimberger E, Ottenjann R (1985). Angiodysplasia in the colon and rectum Endoscopic morphology, localisation and frequency. Endoscopy.

[9] Strate LL, Syngal S (2003). Timing of colonoscopy: Impact on length of hospital stay in patients with acute lower intestinal bleeding. Am J Gastroenterol.

[10] Scheffel H, Pfammatter T, Wildi S, Bauerfeind P, Marincek B, Alkadhi H (2007). Acute gastrointestinal bleeding: Detection of source and etiology with multi-detector-row CT. Eur Radiol.

[11] Zuckerman DA, Bocchini TP, Birnbaum EH (1993). Massive hemorrhage in the lower gastrointestinal tract in adults: Diagnostic imaging and intervention. AJR Am J Roentgenol.

[12] Nicholson ML, Neoptolemos JP, Sharp JF, Watkin EM, Fossard DP (1989). Localization of lower gastrointestinal bleeding using in vivo technetium-99m-labelled red blood cell scintigraphy. Br J Surg.

[13] Browder W, Cerise EJ, Litwin MS (1986). Impact of emergency angiography in massive lower gastrointestinal bleeding. Ann Surg.

[14] Strate L Approach to the adult patient with lower gastrointestinal bleeding.

